# Back from the dead: validity and taxonomic position of *Cotylurus brandivitellatus* (Belogurov, Maksimova et Tolkacheva, 1966) in light of the integrative taxonomy approach

**DOI:** 10.1017/S003118202510067X

**Published:** 2025-09

**Authors:** Julia Gabrysiak, Gerard Kanarek, Beata Rydelek, Sandra Wydra, Grzegorz Zaleśny, Joanna Hildebrand

**Affiliations:** 1 Department of Parasitology, Faculty of Biological Sciences, University of Wrocław, Wrocław, Poland; 2 Ornithological Station, Museum and Institute of Zoology, Polish Academy of Sciences, Gdańsk, Poland; 3 Pomeranian Wildlife Rehabilitation Centre ‘Ostoja’, Pomieczyno, Poland; 4 Department of Invertebrate Systematics and Ecology, Institute of Biology, Wrocław University of Environmental and Life Sciences, Wrocław, Poland

**Keywords:** *Cotylurus*, integrative approach, taxonomy

## Abstract

Accurate species identification is essential for biodiversity research, especially in the field of parasitological systematics. In particular, the incorporation of DNA-based methods in the study of Digenea has transformed taxonomy by allowing for precise species delimitation, clarification of life cycles, and the identification of cryptic diversity. However, to prevent taxonomic misidentification, a growing concern in public sequence databases, these molecular techniques must be supplemented with high-quality morphological data. This study provides an integrative assessment (combining both morphological and molecular data) of *Cotylurus brandivitellatus*, based on adult specimens obtained from naturally infected mute swan (*Cygnus olor*) in Gdańsk Pomerania. The observed morphological characteristics are consistent with the original description of *C. brandivitellatus* and align with the established description of the genus *Cotylurus*. Phylogenetic analysis, utilizing concatenated LSU rDNA and COI mtDNA markers, confirms the distinct taxonomic status of *C. brandivitellatus*. It forms a sister clade with *C. strigeoides*, which is clearly separate from other species within the *Cotylurus* genus. These findings validate the existence of *C. brandivitellatus* and offer new insights into species delineation and evolutionary relationships within *Cotylurus*, highlighting the importance of integrative approaches in trematode systematics.

## Graphical abstract

**Figure:**
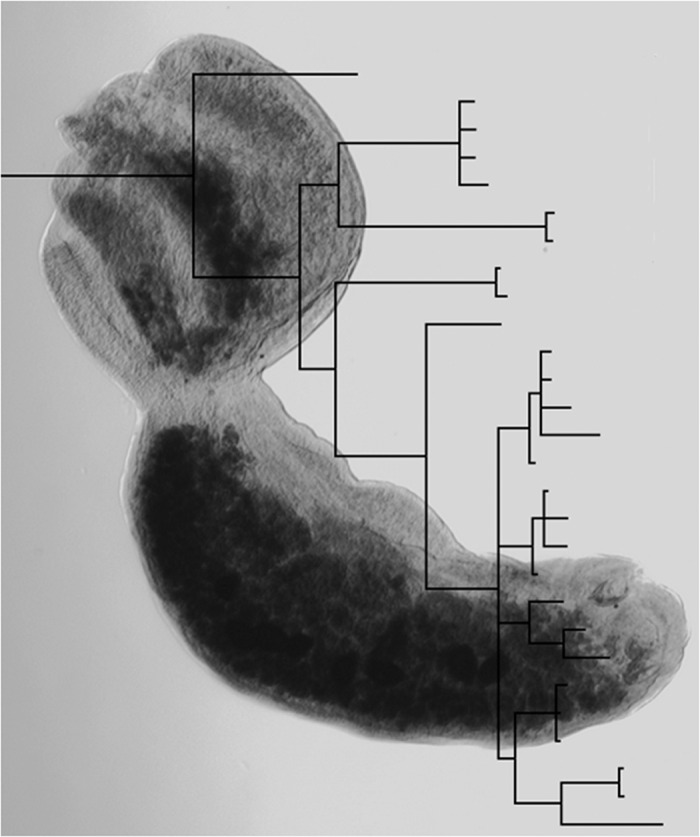

## Introduction

The process of species identification is widely acknowledged as one of the most essential requirements for understanding the diversity, ecology, and evolution of the living world (e.g., Elphick, 2008; Farnsworth et al., 2013; Kürzel et al., 2022). In recent decades, DNA sequencing technologies have become integral to all branches of zoology, enabling precise delimitation and characterization of specific species, as well as establishing their taxonomic positions. Importantly, in the contemporary taxonomy of Digenea, DNA technologies have not only facilitated accurate species delimitation and valuable phylogenetic reconstructions but have also provided a unique means of identifying and differentiating all life stages of trematodes. This capability has greatly enhanced the understanding of trematode life cycles and helped recognize cryptic diversity (e.g., Georgieva et al., 2013a, 2013b; Bray et al., 2022; Pyrka et al., 2022; Valadão et al., 2023). Given these advancements, molecular data have become a ‘must-have’ tool for nearly all ecological and evolutionary research, large-scale biodiversity surveys, and species identification and delineation. However, a significant limitation in using DNA sequences for the taxonomy of Digenea – and zoology in general – is the necessity for precise taxonomic identification of sequenced specimens using conventional morphology-based analysis. This step is crucial to ensure that the obtained sequences are correctly linked to their taxonomic identities. Therefore, molecular techniques should always be combined with high-quality morphological studies to achieve reliable results (e.g., Blasco-Costa et al., 2016; Schwelm et al., 2021; Faltýnková et al., 2022). Additionally, the increasing problem of taxonomic misidentification in public DNA databases, which arises from improperly identified isolates (due to issues such as invalid labelling, poor quality, lack of voucher specimens for further comparison, or the use of non-informative DNA markers in constructed phylogenies), has been widely discussed in contemporary literature (Locke et al., 2015; Kanarek et al., 2016; Steinegger and Salzberg, 2020; Tang, 2020; Bensch et al., 2021; Achatz et al., 2022; Coca-de-la-Iglesia et al., 2024).

Despite certain challenges, the understanding of the genus *Cotylurus* (Szidat, 1928) (Diplostomoidea: Strigeidae) has evolved significantly in recent years. This change has been largely due to the application of molecular methods and an integrative taxonomy approach. *Cotylurus* comprises a relatively small group of highly specialized species that inhabit the intestines and the bursa of Fabricius of water and wading birds (for further details, see Pyrka et al., 2021, and references therein). Established by Szidat in 1928, the genus *Cotylurus* includes strigeid trematodes from avian hosts, characterized by vitellaria that are limited to the opisthosoma and a well-developed genital bulb. *Cotylurus* operates on a 3-host life cycle closely associated with the freshwater environment. The first intermediate hosts, in which the larval stages multiply asexually, are typically pulmonate snails. A variety of pulmonate and prosobranch water snails, as well as leeches, serve as the second intermediate hosts (for further details, see Pyrka et al., 2021, 2022, and references therein). The metacercariae of the tetracotyle type are transmitted to the avian definitive hosts when they ingest the second intermediate host (Sudarikov et al., 2002; Cribb et al., 2003; Blasco-Costa and Locke, 2017).

*Cotylurus* is widely recognized as a valid genus (Niewiadomska, 2002; Heneberg et al., 2018; Pyrka et al., 2021); however, its species composition remains controversial. The absence of easily visible morphological features and the high level of morphological variability observed in adult forms of species have led to numerous taxonomic decisions of questionable validity by various authors (e.g., Dubois, 1968; Sudarikov, 1984). Consequently, this group has been the focus of extensive discussion and ongoing taxonomic changes over time. As a result, the legitimacy of certain species within the genus *Cotylurus* and the true diversity of this genus remain ambiguous and require further confirmation. Among the many species of *Cotylurus*, one with a particularly complicated and still unclear taxonomic status is *Cotylurus brandivitellatus* (Belogurov et al., 1966). This species was originally described based on trematode specimens collected from the intestines of a wide range of ducks (including the northern shoveler [*Spatula clypeata*], garganey [*Spatula querquedula*], greater scaup [*Aythya marila*], and long-tailed duck ([*langula hyemalis*]) sampled in the lower reaches of the Yenisei River, along the coast of the Sea of Okhotsk, and in Kazakhstan (Belogurov et al., 1966).

Initially, Belogurov et al. (1966) placed his species within the genus *Cotylurostrigea* established by Sudarikov (1961) for strigeid trematodes found in Anatid birds. *Cotylurostigea* is characterized by a unique combination of morphological features: the presence of vitellaria in both the prosoma and opisthosoma (a trait typical for the genus *Strigea*) and a well-developed genital bulb (typical for the genus *Cotylurus*). Sudarikov (1961) initially included only one species in this new genus – *Cotylurostrigea raabei* (Bezubik, 1958) – and later added another taxon, *Cotylurostrigea strigeoides* (Dubois, 1958) (e.g., Sudarikov et al., 2002). Over the years, the validity of *Cotylurostrigea* has been widely debated. Dubois (1968) and Odening (1969) considered it a synonym of *Cotylurus*, while Yamaguti (1971) assigned it a subgeneric status within *Strigea*. However, Sudarikov (1984) still regarded *Cotylurostrigea* as a valid taxon. A cladistic analysis based on morphological and ecological features by Zazornova and Sysoev (1993) recognized *Cotylurostrigea* as a synonym of *Cotylurus*, a view later supported by Niewiadomska (2002). Recent molecular studies have further corroborated these conclusions (Pyrka et al., 2021, 2022). Regardless of the varying generic status of *Cotylurostrigea*, the validity and taxonomic position of *C. brandivitellatus* have also been the subject of extensive discussion over the years and remain unresolved. Odening (1969) and Sudarikov (1984) treated this species as valid, while McDonald (1981) and Niewiadomska (2010) suggested that it be considered a synonym of *C. strigeoides*. Thus, the validity and taxonomic position of *C. brandivitellatus* remain contentious and require further detailed studies.

In this study, we present comprehensive morphological and molecular characterization of adults of *C. brandivitellatus*, sourced from the intestines of naturally infected mute swans (*Cygnus olor*) in Gdańsk Pomerania. Our analysis adheres to the recommended ‘best practices’ established by Blasco-Costa et al. (2016) for molecular approaches in trematode systematics. Based on our findings, we assert new insights regarding the validity and position of *C. brandivitellatus*, as well as its implications for the structure of the genus *Cotylurus*. We firmly believe that this study contributes significantly to clarifying the species spectrum within the *Cotylurus* genus through an integrative taxonomy approach, supported by sequences that correlate with the well-documented morphology of adult specimens.

## Materials and methods

### Host sampling, parasite recovery protocol and morphological analysis

Adult trematodes were collected from the intestinal tract of a juvenile mute swan (*Cygnus olor*) specimen weighing approximately 2.5 kg. This swan was received deceased with permission (RDOŚ-Gd-WZG.6401.64.2023.AB.3) from the Pomeranian Wildlife Rehabilitation Centre ‘Ostoja’. The research material consisted of bird specimens that had been euthanized due to severe injuries while providing necessary veterinary assistance to sick and weakened birds from the Gdańsk Pomerania area. The avian hosts were transported to the laboratory immediately after death and then frozen at −25°C for further necropsy. Specimens of *Cotylurus* sp. were identified in the intestine, washed in tap water and preserved in hot 70% ethanol for further morphological and molecular studies. The collected trematodes were initially identified under a stereomicroscope following the original description provided by Belogurov et al. (1966). Some selected trematodes were designated for further molecular studies and were vouchered according to the protocols outlined by Pleijel et al. (2008) and Blasco-Costa et al. (2016). A series of microphotographs were taken using a digital camera. For DNA extraction, a small fragment of the opisthosoma was excised from the selected specimens. The remaining voucher specimen (hologenophore) and other trematode specimens identified as *C. brandivitellatus*, collected from the same hosts (paragenophores), were stained with iron acetocarmine, dehydrated in ethanol, cleared in clove oil, and mounted in Canada balsam for detailed morphological studies. All measurements were taken from the paragenophores stained and mounted in Canada balsam using NIS-Elements D image analysis software. The voucher specimens were deposited in the Natural History Museum of Geneva under number MHNG-PLAT-0159729.

### DNA processing and phylogenetic analyses

DNA was extracted from small piece of hologenophores and selected single alcohol-fixed paragenophores using a commercial kit (DNeasy Blood and Tissue kit; Qiagen, Hilden, Germany), according to the manufacturer’s protocol.

PCR amplification of nuclear 28S and the mitochondrial gene encoding CO1 was carried out using the KAPA2G Robust HotStart ReadyMix (Sigma-Aldrich, St. Louis, MO. USA). Reaction conditions and primers were selected based on the literature and our previous study, i.e., LSU5, digl2 and 1500 R for 28S rDNA, and JB3 and CO1_Rtrema for CO1 (Pyrka et al., 2021). The PCR products were visualised following electrophoresis in a 1% agarose gel and purified using the Exo-BAP kit (EURx) and sequenced directly in both directions using the PCR primers. Contiguous sequences were assembled using Geneious software (Geneious 9.1.8; https://www.geneious.com).

The alignments including newly obtained sequences and closely related representatives of Strigeidae currently available in GenBank were prepared using ClustalW multiple alignment implemented in MegaX (Kumar et al., 2018). Sequences of the partial 28S rDNA gene (1042 bp) and the CO1 fragment (307 bp) were aligned in one concatenated dataset. Phylogenetic analyses were conducted using Bayesian inference criteria as implemented in MrBayes ver. 3.2.7 software (Ronquist et al., 2012). The general time-reversible model with estimates of invariant sites and gamma distributed among-site variation (GTR + I + G) was identified as the best-fitting nucleotide substitution model for each marker independently and the concatenated dataset. The consensus trees were visualized in FigTree ver. 1.4.4 software (Rambaut, ) and annotated in CorelDraw® (Corel Corp., Ottawa, ON, Canada)

## Results

### Morphological description of the voucher material

**Family: Strigeidae** Railliet, 1919

Subfamily: Strigeinae Railliet, 1919

Genus: *Cotylurus* (Szidat, 1928)

- *Cotylurus brandivitellatus* (Belogurov et al., 1966; Odening, 1969)
- Host: *Cygnus olor* Gmelin, 1789, one individual infected with 42 specimens
- Locality: Chmielno, Gdańsk Pomerania, northern Poland (54°19′34″ N, 18°6′2″E)
- Site of the infection: ileum
- Material: 3 hologenophores and 27 paragenophores
- Representative DNA sequences: PX047982 (28S rDNA), PX056574 (CO1 mtDNA)

**Figure 1.**
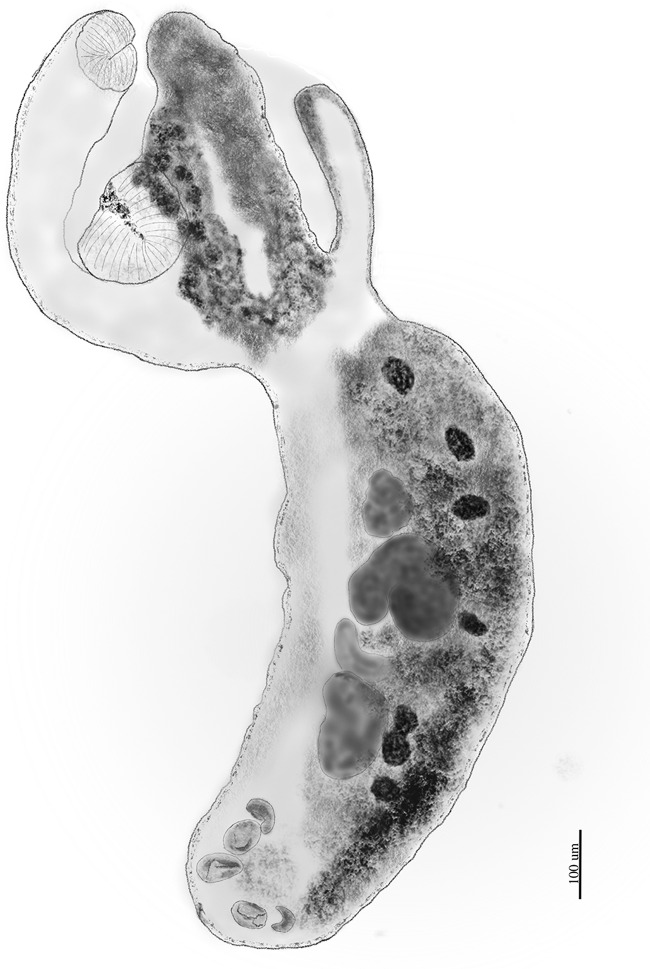
Adult specimen of *Cotylurus brandivitellatus* (host: *Cygnus olor*, locality: Chmielno, Gdańsk Pomerania, Northern Poland).

**Adult** (Fig. 1, Table 1)

**Table 1.** Comparison of selected morphometric characteristics of *C. brandivitellatus, C. magniactebulus, C. lutzi* and related species

Species	*Cotylurus brandivitellatus* (Belogurov et al., 1966; Odening, 1969)	*Cotylurostrigea brandivitellata* (Belogurov et al., 1966)	*Cotylurus magniacetabulus* (Dubois and Angel, 1972)	*Cotylurus lutzi* (Basch, 1969)	*Cotylurus strigeoides* (Dubois, 1958)	*Cotylurus raabei* (Bezubik, 1958)
Author	Present study (*n* = 27)	Belogurov et al. (1966)	Dubois and Angel (1972)	Basch (1969)	Present study (*n* = 17)	Present study (*n* = 10)
Country/Geographic region	Poland/Europe	Russia, Kazakhstan/Asia	Australia	Brasil/South America	Poland/Europe	Poland/Europe
Host	*Cygnus olor*	*Spatula querquedula, S. clypeata, Aythya marila, Clangula hyemalis*	*Cygnus atratus*	*Taeniopygia castanotis, Estrilda troglodytes Lonchura striata* (experimental hosts)	*Anas platyrhynchos*	*Anas platyrhynchos*
Total body length	2024.65−2404.03	1421−2626 (1725)**	2000−2600***	1500−1960 (1661)**	1286.28−1842.43	3277.35−5360.63
	(2207.99 ± 91.34)*				(1587.43 ± 164.13)*	(4425.0 ± 605.6)*
Prosoma length	736.56−870.40	392−998 (650)**	600−710***	400−600 (520)**	489.15−743.21	1296.7−2001.4
	(795.88 ± 37.35)*				(605.32 ± 69.66)*	(1646.6 ± 233.2)*
Prosoma width	699.66−869.26	548−945 (731)**	740−860***	440−700 (606)**	424.67−581.27	1399.4−2277.7
	(769.68 ± 46.91)*				(515.56 ± 45.40)*	(1797.4 ± 300.2)*
Opisthosoma length	1308.00−1576.57	893−1628 (1177)**	1340−1650***	990−1350 (1141)**	721.51−1173.48	1980.68−3359.23
	(1406.88 ± 77.67)*				(982.19 ± 112.73)*	(2778.35 ± 396.62)*
Opisthosoma width	490.55−644.63	515−872 (594)**	530−670***	400−530 (495)**	381.64−607.04	933.65−1592.74
	(574.48 ± 4 0.15)*				(501.49 ± 63.71)*	(1338.09 ± 196.26)*
Prosoma to opisthosoma length ratio	1:1.53−2.05	1: 1.7−2.1 (1.66)**	1: 2−2.6***	1:2.19−2.45****	1:1.28−2.08	1:1.51−2.01
	(1.77 ± 0.11)*				(1.63 ± 0.18)*	(1.69 ± 0.15)*
Oral sucker length	136.42−159.02	112−176 (176)**	105−127***	118−145 (133)**	101.16−120.09	165.66−283.52
	(148.94 ± 5.99)*				(110.88 ± 5.46)*	(223.51 ± 39.51)*
Oral sucker width	120.25−153.66	100−151 (147)**	130−155***	118−145 (133)**	98.08−130.73	155.32−225.57
	(135.54 ± 9.76)*				(115.27 ± 9.75)*	(193.87 ± 26.92)*
Ventral sucker length	186.21−241.49	168−206 (193)**	180−240***	170−188 (173)**	112.58−139.28	222.92−311.66
	(207.59 ± 13.78)*				(123.18 ± 7.0)*	(270.70 ± 36.55)*
Ventral sucker width	153.01−229.41	147−189 (181)**	160−190***	170−188 (173)**	106.48−147.85	232.82−325.28
	(191.39 ± 17.80)*				(126.88 ± 12.01)*	(284.72 ± 38.59)*
Ventral sucker/oral sucker length ratio	1:1.17−1.68	1:1.09	1.35−1.74 (1.47)**	1:1.33−1.44****	1:1.02−1.25	1:1.10−1.35
	(1.40 ± 0.12)*				(1.12 ± 0.07)*	(1.20 ± 0.10)*
Pharynx length	75.23−110.08	56−130 (97)**	80−95***	60−80 (70)**	72.99−78.84	143.06−193.95
	(94.26 ± 8.60)*				(75.91 ± 2.93)*	(164.33 ± 18.09)*
Pharynx width	71.18−92.20	50−100 (88)**	64−75***	9)	52.51−57.56	108.93−187.44
	(80.55 ± 5.46)*				(55.04 ± 2.53)*	(141.25 ± 26.66)*
Ovary length	120.10−199.25	105−179 (160)**	130−160***	90−150 (119)**	99.37−132.97	258.73−355.02
	(149.67 ± 19.43)*				(113.45 ± 9.40)*	(317.75 ± 34.27)*
Ovary width	104.65−169.45	134−199 (134)**	175−210***	90−150 (119)**	92.12−141.41	280.92−379.45
	(138.85 ± 17.07)*				(122.35 ± 13.71)*	(317.62 ± 37.04)*
Anterior testes length	193.32−263.70	179−452 (290)**	210−350***	104−182 (138)**	180.74−278.99	538.51−1117.42
	(224.87 ± 22.03)*				(200.42 ± 28.21)*	(836.68 ± 163.61)*
Anterior testes width	109.73−235.97	159−431 (210)**	275−320***	143−260 (198)**	125.63−187.05	566.06−1139.25
	(186.18 ± 37.75)*				(156.29 ± 17.81)*	(758.84 ± 191.77)*
Posterior testes length	169.63−272.86	179−420 (277)**	210−320***	104−182 (138)**	181.79−270.12	500.86−1209.08
	(212.87 ± 29.00)*				(216.66 ± 27.19)*	(913.89 ± 204.76)*
Posterior testes width	125.77−223.22	190−530 (256)**	265−300***	104−182 (138)**	149.79−211.93	521.37−1076.82
	(163.86 ± 27.54)*				(172.08 ± 17.80)*	(831.25 ± 165.62)*
Anterior/posterior testes length ratio	1:0.75−1.39	–	–	1:1****	1:0.94−1.31	1:0.93 1.24
	(0.94 ± 0.18)*				(1.10 ± 0.10)*	(1.09 ± 0.09)*
Egg length	70.94−96.52	79.8−96.6***	84−99***	–	72.79−96.11	118.50−138.43
	(85.09 ± 7.50)*				(84.71 ± 6.39)*	(129.52 ± 5.92)*
Egg width	46.07−62.95	50.4−75.6***	58−72***	–	43.01−58.75	69.98−87.27
	(55.11 ± 4.47)*				(52.65 ± 4.33)*	(77.88 ± 5.65)*
Pre-ovary distance in opisthosoma	212.91−339.44	–	–	–	27.53−76.03	122.07−562.51
	(268.83 ± 28.91)*				(53.89 ± 16.47)*	(270.64 ± 167.64)*
Pre-ovary distance in opisthosoma [% of opisthosoma length]	15.90−22.67	–	–		2.85−7.29	4.12−16.75
	(19.10 ± 1.60)*				(5.19 ± 1.61)*	(9.8 ± 5.1)*
Post-testicular distance in opisthosoma	327.04−467.40	–	–	–	212.91−374.67	344.67−649.82
	(398.54 ± 35.04)*				(292.64 ± 50.21)*	(499.44 ± 112.59)*
Post-testicular distance in opisthosoma [% of opisthosoma length]	24.25−31.96	–	–	–	25.63−33.72	14.02−21.13
	(28.39 ± 2.12)*				(30.37 ± 3.21)*	(18.41 ± 2.54)*

* Min–max (mean ± SD).

** Min–max (mean).

*** Min–max.

**** Ratio calculated on base of literature data.

The following information are based on 27 paragenophores stained with iron acetocarmine and mounted on slides in Canada balsam. All measurements are expressed in micrometres (min-max; mean ± SD) unless otherwise specified. The specimens are mounted laterally, ensuring that the prosoma and opisthosoma organ widths match their dorso-ventral diameters.

Body distinctly bipartite, slightly curved, total length 2024.65–2404.03 (2207.99 ± 91.34). Tegument smooth. Prosoma cupuliform, hemispherical to spheroidal 736.56–870.40 (795.88 ± 37.35) length and 699.66–869.26 (769.68 ± 46.91) width, maximum width at the level of the ventral sucker. Prosoma well separated from subcylindrical and slightly arched opisthosoma, 1308.00–1576.57 (1406.88 ± 77.67) length and 490.55–644.63 (574.48 ± 40.15) width. The length ratio of the prosoma to the opisthosoma is 1:1.53–2.05 (1.77 ± 0.11). Oral sucker muscular, terminal and elongate-oval, 136.42–159.02 (148.94 ± 5.99) length and 120.25–153.66 (135.54 ± 9.76) width. Ventral sucker muscular and elongate-oval, larger than the oral sucker, usually positioned at the mid-level of the prosoma, 186.21–241.49 (207.59 ± 13.78) length and 153.01–229.41 (191.39 ± 17.80) width. The oral to ventral sucker length ratio is 1:1.17–1.68 (1.40 ± 0.12). No prepharynx observed. The pharynx muscular weakly and slightly oval, 75.23–110.08 (94.13 ± 8.63) length and 71.18–92.20 (80.82 ± 5.61) width. The oesophagus, intestinal bifurcation, and caeca in the prosoma not observed. The caeca in the opisthosoma poorly visible and asymmetrical. The holdfast organ consists of 2 large lobes with a deep slit. The ovary oval to reniform, pretesticular, 120.10–199.25 (147.79 ± 17.87) length and 104.67–169.45 (138.85 ± 17.07) width, positioned 212.91–339.44 (268.83 ± 28.91) from the anterior end of the opisthosoma. The pre-ovary distance in the opisthosoma relatively large, occupying 15.90–22.67 (19.10 ± 1.60)% of the total opisthosoma length. Laurer’s canal and Mehlis gland not visible. Two testes, post-ovarian, large, tandem and extended-oval, usually bilobed with asymmetrical lobes. Slightly larger anterior testis 193.32–263.70 (224.87 ± 22.03) length and 109.73–235.97 (186.18 ± 37.75) at the widest point. The posterior testis 169.63–272.86 (212.87 ± 29.00) length and 125.77–223.22 (163.86 ± 27.54) width. The length ratio of the anterior to posterior testis is 1:0.75–1.39 (0.94 ± 0.18). The post-testicular region of the opisthosoma measures 327.04–467.40 (397.9 ± 35.35), covering 24.25–31.96 (28.39 ± 2.12)% of the opisthosoma length. The seminal vesicle not observed. The vitellaria dense and follicular, containing numerous vitelline follicles, present in both the prosoma and opisthosoma, with follicles in the prosoma extending anteriorly into the distal parts of the lobes of the holdfast organ at various lengths, typically asymmetrically. Vitellaria absent in the ‘neck’ region and do not occur in the parenchyma tissue between the prosoma and opisthosoma. In the opisthosoma, the vitelline fields extend from the pre-ovarian region to the mid-level of the genital bulb. The genital bulb well-expressed, clearly delimited from the surrounding parenchyma. The excretory vesicle and excretory pore not observed. The uterus extends anterior to the ovary, ventral to the gonads, containing 15–32 (25.11 ± 4.86) eggs. The eggs oval, 70.94–96.52 (86.09 ± 7.50) length, 46.07–62.95 (55.11 ± 4.47) width.

### Remarks

**Figure 2.**
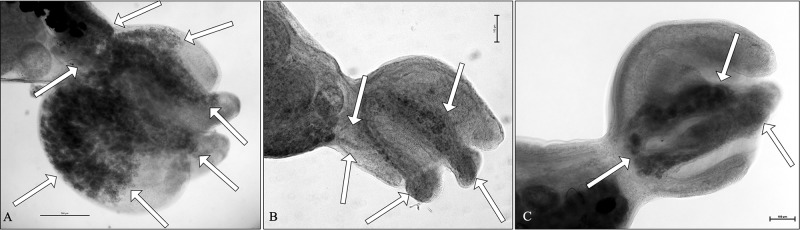
Comparison of vitellaria in prosoma of 3 *Cotylurus* species from anatid avian hosts: (A) – *Cotylurus raabei*; (B) – *Cotylurus strigeoides*; (C) – *Cotylurus brandivitellatus.* Arrows indicate the distribution of the vitelline follicles in the prosoma.

The analysed material aligns with the diagnosis of the genus *Cotylurus*, as described by Niewiadomska (2002); moreover, the morphology of the presented materials corresponds well with the brief description of *Cotylurostrigea brandivitellata* as provided by Belogurov et al. (1966). This includes an elongate, subcylindrical, slightly curved opisthosoma, a cupuliform, hemispherical to spheroidal prosoma with 2 lobes of the holdfast organ, as well as well-developed suckers and pharynx. The large lobed testes and, importantly, the vitelline follicles located mainly in the opisthosoma – which characteristically extend anteriorly into 2 lobes of the holdfast organ in the prosoma (Figs. 1 and 2) – are also significant features. From a morphometric perspective, most mean body dimensions of the recently analyzed material from a mute swan are slightly larger compared to the originally described specimens of *C. brandivitellatus* from a wide range of duck species. However, the width of the opisthosoma, dimensions of the acetabulum and pharynx, as well as the measurements of the testes, are slightly larger in the original description (Table 1). Notably, the range of variability in measurements from the originally described trematodes is significantly broader than that of the recently studied specimens (Table 1). Additionally, the mean ratio of sucker lengths in the description by Belogurov et al. (1966) is lower than that of the analyzed material (1:1.09 vs. 1:1.40 – Table 1). Despite these differences, we believe that the generally well-corresponding morphology – especially the structure of vitellaria in the prosoma – along with the similarity in dimensions of the body, internal organs, and certain morphological indices (such as the ratio of prosoma to opisthosoma length) supports the conclusion that our material represents *C. brandivitellatus*.

The morphology of *C. brandivitellatus* is characterized by a unique structure of vitelline follicles in the prosoma, which closely resembles 2 other species of *Cotylurus*, i.e. *C. lutzi* (Basch, 1969) and *C. magniacetabulus* (Dubois and Angel, 1972). *C. lutzi* was described based on trematode specimens obtained experimentally from tetracotyle, which were collected from the naturally infected snail, *Biomphalaria glabrata,* in Bahia, Brazil and fed to atypical avian passeriform hosts: the Sunda zebra finch (*Taeniopygia guttata*), black-rumped waxbill (*Estrilda troglodytes*) and white-rumped munia (*Lonchura striata*) (Basch, 1969).

The adult trematodes obtained in this study, although similar in the structure of vitellaria in the opisthosoma and sucker length ratio (1.35–1.74 in *C. lutzi* vs. 1.17-1.68 in *C. brandivitellatus*), differ significantly from *C. brandivitellatus* specimens in several aspects: smaller body size and dimensions of all internal organs, round (not elongated) testes, and a significantly larger prosoma to opisthosoma length ratio (1:2.19–2.45 vs. 1:1.53–2.05) (Table 1). Notably, adult specimens of *C. lutzi* have not been recorded in definitive avian hosts since their description, leaving their true morphological and morphometric variability unknown. The other species, *C. magniacetabulus*, was characterized based on several trematode specimens collected from the ‘lower intestine’ of a naturally infected black swan (*Cygnus atratus*) in Australia (Dubois and Angel, 1972) Besides the evident similarity in the structure of vitellaria in the prosoma, *C. magniacetabulus* shows similarities in general morphology with the recently analysed specimens of *C. brandivitellatus*. However, the 2 species differ in a few body dimensions (Table 1). The main differences between the trematode specimens analysed and the original description of *C. magniacetabulus* include larger total body length, shorter prosoma and longer opisthosoma in *C. magniacetabulus* (resulting in a slightly higher prosoma to opisthosoma length ratio: 1:1.53–2.05 vs. 1:2–2.6), larger testes, wider ovary and larger egg width (Table 1). Interestingly, since the description, *Cotylurus* specimens identified on the basis of morphology as *C. magniacetabulus* have been recorded in Anatidae in South America (Padilla-Aguilar et al., 2020a, 2020b), a region typical for *C. lutzi*. Despite these differences, we believe that the morphology of *C. brandivitellatus*, particularly the arrangement of vitelline follicles in the prosoma, is uniquely consistent within the genus *Cotylurus*, aligning with further descriptions of *C. lutzi* and *C. magniacetabulus.* The existence or absence of vitelline follicles in the prosoma, along with their structure, is widely regarded as one of the most important and relatively constant morphological features crucial for the precise identification of particular species within *Cotylurus* (e.g., Dubois, 1968; Sudarikov, 1984). Therefore, it seems unlikely that the identical structure of vitellaria in the prosoma of these 3 species is merely coincidental. When considering the broader implications, the observed differences in morphology and morphometry among *C. brandivitellatus, C. lutzi* and *C. magniacetabulus* are, in our opinion, not significant and may be results of the origins of the voucher specimens (especially the experimental infections of non-specific passeriform hosts in the case of *C. lutzi*) and geographic variations. Literature extensively describes phenotypic variations in Digenea caused by the identity of the host (‘host-induced variation’) (e.g., Stunkard, 1957; Nolan and Cribb, 2005; Hildebrand et al., 2015). Notably, the original descriptions of *C. lutzi* and *C. magniacetabulus* (Basch, 1969; Dubois and Angel, 1972) do not include a differential diagnosis with *C. brandivitellatus*. Given these observations, we are convinced that the 3 taxa are conspecific; however, final confirmation of this hypothesis requires morphological and molecular analyses of *Cotylurus* specimens from Australia and South America.

### Phylogenetic results

**Figure 3.**
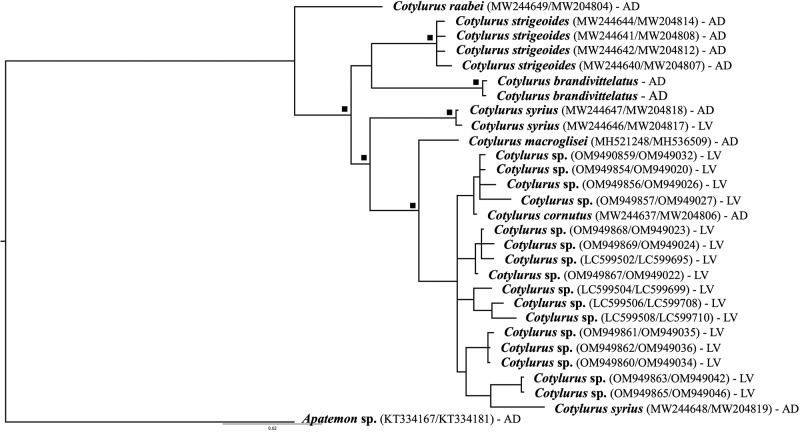
The phylogenetic reconstruction of *C. brandivitellatus* based on the concatenated COI mtDNA and 28S rDNA markers. The analysis was performed by the use of Bayesian inference, square symbol indicates posterior probability greater than 90%. AD – adult; LV – larvae.

Phylogenetic analysis supports the hypothesis of independent taxonomic position and validity of *C. brandivitellatus*. In the case of 28S rDNA, *C. brandivitellatus* forms an independent lineage sister to *C. strigeoides* (Figure 3) with a sequence similarity of 99.4% (8 nucleotide difference). Phylogenetic analysis based on mtDNA COI even more clearly shows the independent status of *C. brandivitellatus*, also with *C. strigeoides* as a sister lineage to *C. brandivitellatus* (Figure 3). The genetic similarity of the sequences is at the level of 90%. Obtained results also confirmed separate position of *C. raabei* within genus *Cotylurus* (Figure 3).

## Discussion

Despite extensive efforts in recent years, the actual diversity, phylogenetic relationships and species composition of the genus *Cotylurus* are still not fully established. Recent study, which combine morphological and molecular analyses of adult *Cotylurus* collected from avian hosts in Central Europe, have revealed an unexpectedly high level of molecular diversity within morphologically well-defined species. This includes the unclear status of the typical species of the genus *Cotylurus*, such as *C. cornutus* (Rudolphi, 1808; Szidat, 1928), the polyphyletic nature of *C. syrius* Dubois, 1934 and the distinct species status of *C. raabei* (Bezubik, 1958; Pyrka et al., 2021). Further research focusing on molecular analyses of tetracotyle metacercariae of *Cotylurus*, collected from snail second intermediate hosts, has again shown high molecular diversity and clear evidence of cryptic taxa. Several putative novel species lineages have also been identified. These findings reaffirm the polyphyletic nature of *C. syrius* (with 3 separate molecular species-level lineages) and *C. cornutus* (with 4 separate molecular species-level lineages). This strongly suggests that these taxa, in fact, consist of a complex of species (Pyrka et al., 2022). Notably, 2 divergent phylogenetic and ecological lineages within *Cotylurus* were demonstrated – one utilizing leeches and the other freshwater snails as second intermediate hosts, which differ significantly in their life history strategies (Pyrka et al., 2021, 2022). Despite these advancements in understanding the diversity, morphological and molecular variability, and phylogeny of *Cotylurus*, several issues regarding the taxonomy, biology, and ecology of these trematodes remain unresolved.

Our results obtained from an integrative taxonomy approach shed new light on the current understanding of the phylogeny and diversity of the genus *Cotylurus*, leading to the confirmation of the validity and phylogenetic affiliations of *C. brandivitellatus*. In our opinion, the new material of adult *C. brandivitellatus*, despite some morphometric differences, corresponds well with the original description. The unique morphology of the analyzed trematode specimens – especially the structure of vitelline follicles in the prosoma, as well as the body and internal organ measurements – generally aligns with the original description and the body dimensions provided by Belogurov et al. (1966). The observed variability in specific morphometric features between the recently collected material and the original description is likely related to geographical differences (Europe and Asia), the phylogenetic distance, and the body dimensions of the avian hosts. It is noteworthy that since the description of *C. brandivitellatus*, it has rarely been recorded in the former Soviet Union across various anatid avian hosts, including the red-throated loon (*Gavia stellata*) and the short-billed dowitcher (*Limnodromus griseus*) (Sudarikov, 1984), as well as in Poland in mute swans (Sulgostowska, 1972). The recent findings of this trematode species in a juvenile, flightless specimen of mute swan clearly indicate a local origin of the infection, suggesting that the life cycle of *C. brandivitellatus* is completed within the aquatic ecosystems of Central Europe.

The phylogenetic analysis confirms that *C. brandivitellatus* is a valid species within the genus *Cotylurus*, refuting earlier claims by McDonald (1981) and Niewiadomska (2010) of it being conspecific with *C. strigeoides*. The analysis also highlights close affinities between these 2 taxa. Historically, *C. brandivitellatus* and *C. strigeoides*, along with *C. raabei*, were classified as *Cotylurostrigea* by Sudarikov (1961, 1984). He argued that their unique features warranted a separate genus. However, recent molecular studies indicate that *Cotylurostrigea* is a junior synonym of *Cotylurus* (Pyrka et al., 2021). Our findings support this matter, highlighting the evolutionary significance of the distribution of vitelline follicles in the prosoma. Additionally, *C. raabei* is confirmed as distinct from *C. brandivitellatus* and *C. strigeoides* (Figure 3), contributing to a clearer understanding of *Cotylurus* phylogeny. Niewiadomska’s (1971) hypothesis posits that early divergent *Cotylurus* forms had vitelline follicles in both the prosoma and opisthosoma, while evolutionary derived forms exhibit them only in the opisthosoma. This aligns with the observation that *C. raabei* is the earliest divergent morphological form, with distinct vitellaria extending from the opisthosoma to the prosoma. Moreover, *C. lutzi* and *C. magniacetabulus* exhibit similar vitelline follicles patterns to *C. brandivitellatus*, suggesting potential conspecific status that deserves further investigation. A thorough study combining morphological, molecular, and ecological data, especially with a focus on non-European specimens, is needed. We believe this will yield valuable insights into the diversity and relationships within the *Cotylurus* genus.
